# Long-term outcomes of cementless femoral stem revision with the Wagner cone prosthesis

**DOI:** 10.1186/s13018-021-02457-8

**Published:** 2021-06-11

**Authors:** Kyung-Soon Park, Sheng-Yu Jin, Jun-Hyuk Lim, Taek-Rim Yoon

**Affiliations:** Department of Orthopedic Surgery, Center for Joint Disease, Chonnam National University Hwasun Hospital, 322 Seo Yang-Ro, Hwasun-Eup, Hwasun-Gun, Gwangju, Jeonnam 519-809 Republic of Korea

**Keywords:** Revision total hip arthroplasty, Cementless femoral stem, Wagner cone prosthesis, Stress shielding

## Abstract

**Background:**

The procedure of femoral stem revision is challenging, and bone conservation with less stress shielding is a mandatory effort in these cases. Although there are several reports of stem revision with stems designed for primary total hip arthroplasty (THA), there is no report on stem revision with the Wagner cone prosthesis.

**Methods:**

Between 1996 and 2008, 41 hips of 41 consecutive patients were subjected to femoral revision THA using the Wagner cone prosthesis. The mean age during revision surgery was 56.1 years, and the mean follow-up period was 14.8 years. The clinical results were evaluated, and the femoral component was assessed radiologically.

**Results:**

The results showed that the average period from the first operation to revision THA was 8.0 years. Additionally, the mean Harris hip score improved from 52 points preoperatively to 83 points at the final follow-up. All stems showed bone integration in the radiological evaluation. A subsidence of more than 5 mm was observed in 3 out of 28 (10.7%) femoral stems. Two patients needed an acetabular revision for acetabular cup loosening during the follow-up period. Furthermore, one patient had recurrent dislocation and had to undergo revision surgery for soft tissue augmentation.

**Conclusions:**

We achieved favorable clinical and radiological long-term outcomes in femoral stem revision using the Wagner cone prosthesis. This cementless femoral stem could be an option for femoral stem revision in cases with relatively good bone stock.

## Background

Due to the increasing life-expectancy and the increasing trend of performing total hip arthroplasty (THA) even in young individuals and physically active patients, the number of THAs has risen rapidly, and the revision and re-revision rates are expected to increase proportionally. It is expected that the proportion of revision THAs will increase by 137% between 2005 and 2030 [1].

Revision after failure of femoral components may be technically demanding due to the loss of the periprosthetic bone stock. Moreover, multiple surgeries can result in significant bone and soft tissue deficits. The cemented prosthesis is not a good choice for femoral stem revision; Edward et al. [2] reported that the cemented revision stem showed a higher rate of radiographic failure as compared to the cementless stem in a 20-year follow-up.

Hence, cementless stem revision is the preferred choice in femoral stem revision. Different kinds of cementless stems can be chosen based on the femoral bone loss, such as a proximal porous-coated stem [3], cylindrical stem, extensively porous-coated stem [4], or tapered fluted stem [5].

However, a cementless femoral stem could result in stress shielding issues. Increased length of the femoral stem is associated with a high risk of stress shielding [6]. Alternatively, the use of modular revision stems allows the surgeon to customize each component according to the intraoperative needs, owing to the independent preparation of the proximal and distal femur to optimize the femoral fill and fit, thus avoiding stress shielding. However, even though the modular revision stems can provide more stable fixation, they are usually bulky, which could lead to host bone loss during surgery. Additionally, they are expensive and need technical surgical skills to perform the procedure perfectly for the benefit of the patient and improved functional outcomes.

The Wagner cone prosthesis (Zimmer-Biomet, Warsaw, IN, USA) was designed for use in primary THA. It has a tapered design to provide primary stability with 8 ribs, which ensure increased rotational stability. This prosthesis was introduced in 1995, and its design had been changed once in 2005 since then. Fundamentally, it is still used by many surgeons in THA. This stem can provide distal fixation. Even if the patient presents with a severe bone defect, such as septic hip or developmental dysplasia of hip sequelae, it provides favorable clinical outcomes.

There are several reports on various cementless primary THA stems used in femoral stem revision [5, 7–13], but not on the use of the Wagner cone prosthesis. This study aimed to evaluate the long-term clinical and radiographic outcomes of femoral stem revision using the Wagner cone prosthesis in femoral stem revision.

## Materials and methods

This study evaluated 41 hips of 41 consecutive patients subjected to femoral revision THA by a single surgeon using the Wagner cone prosthesis through a posterolateral approach between 1996 and 2008. Among them, 8 patients were lost to follow-up of less than 10 years, and 5 patients died from other causes unrelated to THA. The remaining 28 patients were followed up for more than 10 years. The mean age of the patients during the revision surgery was 56.1 years, and the mean follow-up period was 14.8 (10.1–20.9) years.

The causes of primary THA were osteonecrosis of the femoral head in 18 hips, osteoarthritis in 5 hips, femoral neck fracture in 3 hips, and rheumatoid arthritis in 2 hips. Among the 28 hips, an acetabular cup revision was performed simultaneously in 14 hips, isolated stem revision in 9 hips, and a liner exchange at the time of stem revision in 5 hips. The initial femoral stems were listed as Autophor (Osteo AG, Selzach, Switzerland) in 10 hips, PFC (Johnson & Johnson, Raynham, MA, USA) in 6 hips, CLS (Zimmer, Winterthur, Switzerland) in 6 hips, and other cemented stems in the remaining 6 hips (Table 1).

**Table 1:** Demographic details and preoperative status of patients

Demographic data	
Male/female	21/7
Age (years)	56.1 (range, 40-76)
F/U duration (years)	14.8 (range, 10.1-20.9)
Mean period from 1st to revision (years)	8.0 (range, 4.2-9.2)
Indication for THA	
Osteonecrosis of the femoral head	18
Osteoarthritis	5
Femoral neck fracture	3
Rheumatoid arthritis	2
Procedure for revision THA	
Stem and cup revision	14
Isolated stem revision and liner change	9
Isolated stem revision	5
Femoral stems before revision	
Autophor (Osteo AG, Selzach, Switzerland)	10
PFC (Johnson & Johnson, Raynham, MA, USA)	6
CLS (Zimmer, Winterthur, Switzerland)	6
Other cemented stems	6

*THA* total hip arthroplasty

All patients were evaluated clinically and radiologically. The clinical results were graded using the Harris hip scores (HHS) [14]. The radiographic evaluation was performed by comparing the patients’ immediate postoperative radiographs and last follow-up radiographs. In order to evaluate the femoral component, the proximal aspect of the femur was divided into 7 zones, as described by Gruen et al. [15]. The radiolucent lines observed at the bone-implant interfaces were evaluated and measured on both the anteroposterior and lateral radiographs of the proximal aspect of the femur. Additionally, a migration analysis was performed by measuring the femoral component vertical subsidence, as described by Callaghan et al. [16]. The femoral component vertical subsidence was defined as a change in the distance from the landmark of the femoral component to the most proximal point of the greater trochanter. Generally, a difference of 5 mm or more in the vertical direction between the immediate postoperative and follow-up measurements indicated the presence of vertical subsidence. All the radiographs were assessed for loosening, stable fibrous fixation, and bone ingrowth around the porous proximal sleeve of the prosthesis using the criteria described by Engh et al. [17]. The classification system by Paprosky et al. [18] was used to evaluate the preoperative bone loss, which is based on the location and extent of the femoral bone loss. The heterotopic bone formation was graded using the classification system by Brooker et al. [19]. The degree of stress shielding was assessed using Engh’s classification [20] by comparing the immediate postoperative radiographs and the last follow-up radiographs. Moreover, evaluations were performed at each follow-up to identify any complications.

The data were analyzed for statistical significance using the chi-square test for categorical variables and the student’s *t* test for continuous variables; *P* values ≤0.05 were considered statistically significant. All statistical analyses were performed using the Statistical Product and Service Solutions software version 25.0 (SPSS, Chicago, IL). We performed a 10-year and expected 15-year survival analysis using the Kaplan–Meier technique with 95% confidence intervals.

## Results

The average period from the first operation to revision THA was 8.0 years, and component aseptic loosening was an indication for surgery. Based on the Paprosky classification of femoral bone loss, there were 10 hips of type IIa, 7 hips of type IIb, 8 hips of type IIc, 2 hips of type IIIa, and 1 hip of type IIIb (Table 2).

**Table 2:** Radiographical results

Parameters	Number of hips (%)
**Preoperative femoral bone defect (Paprosky classification)**	
IIa	10 (35.7)
IIb	7 (25.0)
IIc	8 (28.5)
IIIa	2 (7.1)
IIIb	1 (3.6)
IIIc	0 (0)
**Vertical subsidence**	Average 1.67 mm (0~10mm)
0-3 mm	19 (67.8)
3-5 mm	6 (21.4)
>5 mm	3 (10.7)
**Stability by Engh method**	
Stable osteointegration	26 (92.8)
Stable fibrous fixation	1 (3.5)
Unstable fibrous fixation	1 (3.5)
**Radiolucentline (Gruen zone)**	
I	11 (39.2)
II	3 (10.7)
III	0 (0)
IV	0 (0)
V	0 (0)
VI	2 (7.1)
VII	7 (25.0)
**Heterotopic bone formation**	
Grade I	2 (7.1)
Grade II	2 (7.1)
**Stress shielding**	
Grade I	5 (17.9)
Grade II	2 (7.1)
Grade III	0 (0)

The stem sizes of the Wagner cone prosthesis varied between 16 and 23. For the femoral bone loss, morselized allograft bone grafting was performed in 11 hips of types IIc, IIIa, or IIIb of the Paprosky classification. Additionally, for the prevention of femoral fracture during stem insertion, prophylactic femoral wiring was performed in 8 hips.

The mean HHS improved from 52 (range, 36–62) pre-operatively to 83 (67–98) at the final follow-up. It was noted that there were 13 patients with excellent rating, 10 with good rating, and 4 with fair rating as per the condition results. Only one patient underwent re-revision surgery for acetabular loosening and showed poor results.

**Fig. 1:**
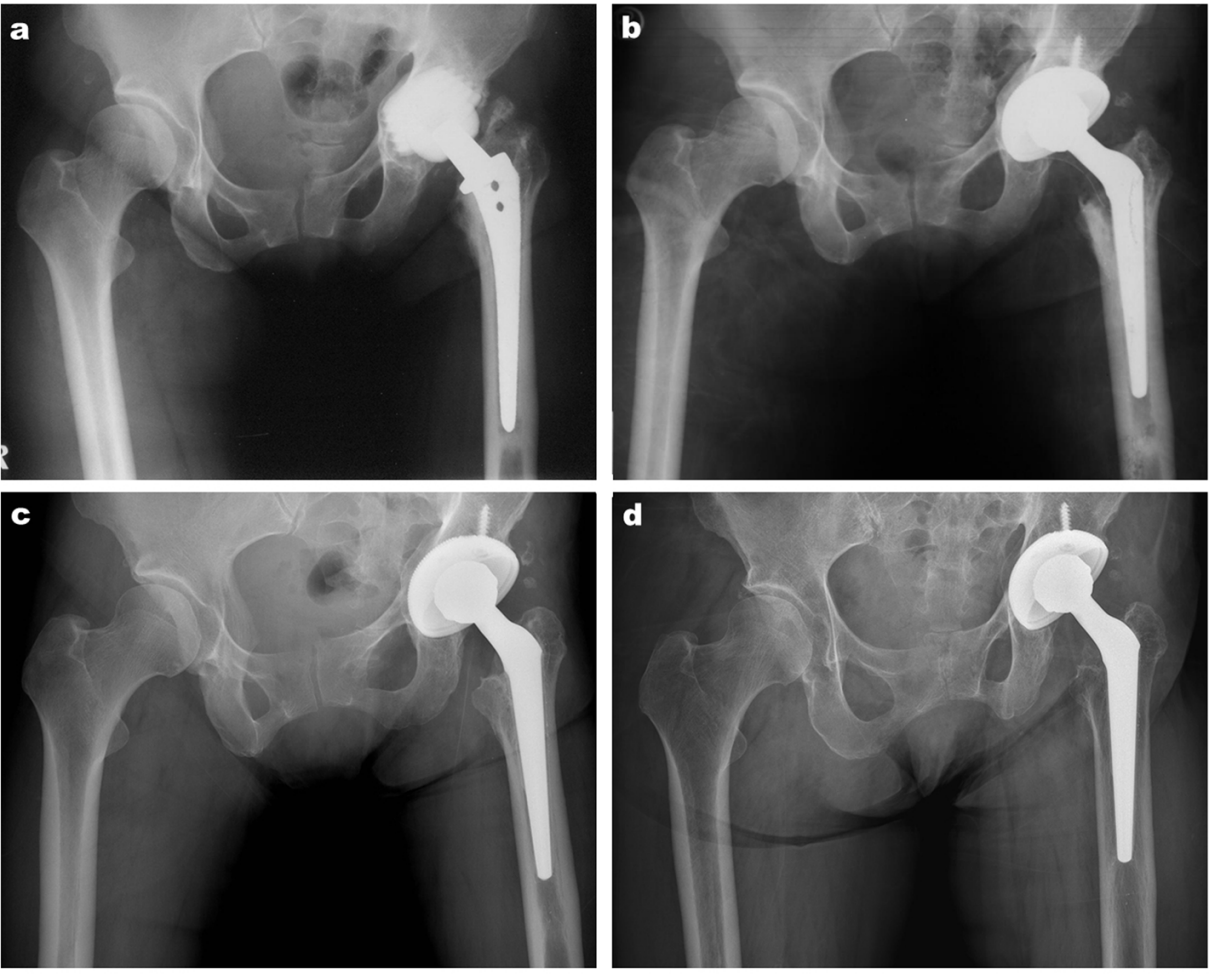
**a** Preoperative radiograph of a 57-year-old woman with aseptic loosening of the Autophor femoral stem. **b** Immediate postoperative radiograph of the left hip: revision was performed using the Wagner cone stem and cementless acetabular cup. **c** Anteroposterior radiographic view of the left hip 3 years after the revision total hip arthroplasty (THA). **d** Anteroposterior radiographic view of the left hip 20 years after the THA shows grade II stress shielding in Gruen zones 1 and 7

All 28 (100%) femoral stems showed bone integration radiologically, and 3 of 28 (10.7%) femoral stems showed subsidence of more than 5 mm. Partial resorption of the calcar femoris with signs of grade I stress shielding was observed in 5 hips (17.9%). Bone resorption in the proximal portion of the femur (Gruen zones 1 and 7), regarded as grade II stress shielding, occurred in 2 hips (7.1%) (Fig. 1). There was no sign of stress shielding in the remaining 21 hips (75%) (Table 2).

Two patients needed acetabular cup revision due to acetabular aseptic loosening during the follow-up period. One patient complained of anterior thigh pain, and one patient had recurrent dislocation and underwent revision surgery for soft tissue augmentation. Additionally, one patient underwent internal fixation for the non-traumatic fracture of the greater trochanter, which was detected during the follow-up. There were no cases presenting periprosthetic fracture, infection, additional stem revision, deep vein thrombosis, or neurovascular injury.

**Fig. 2:**
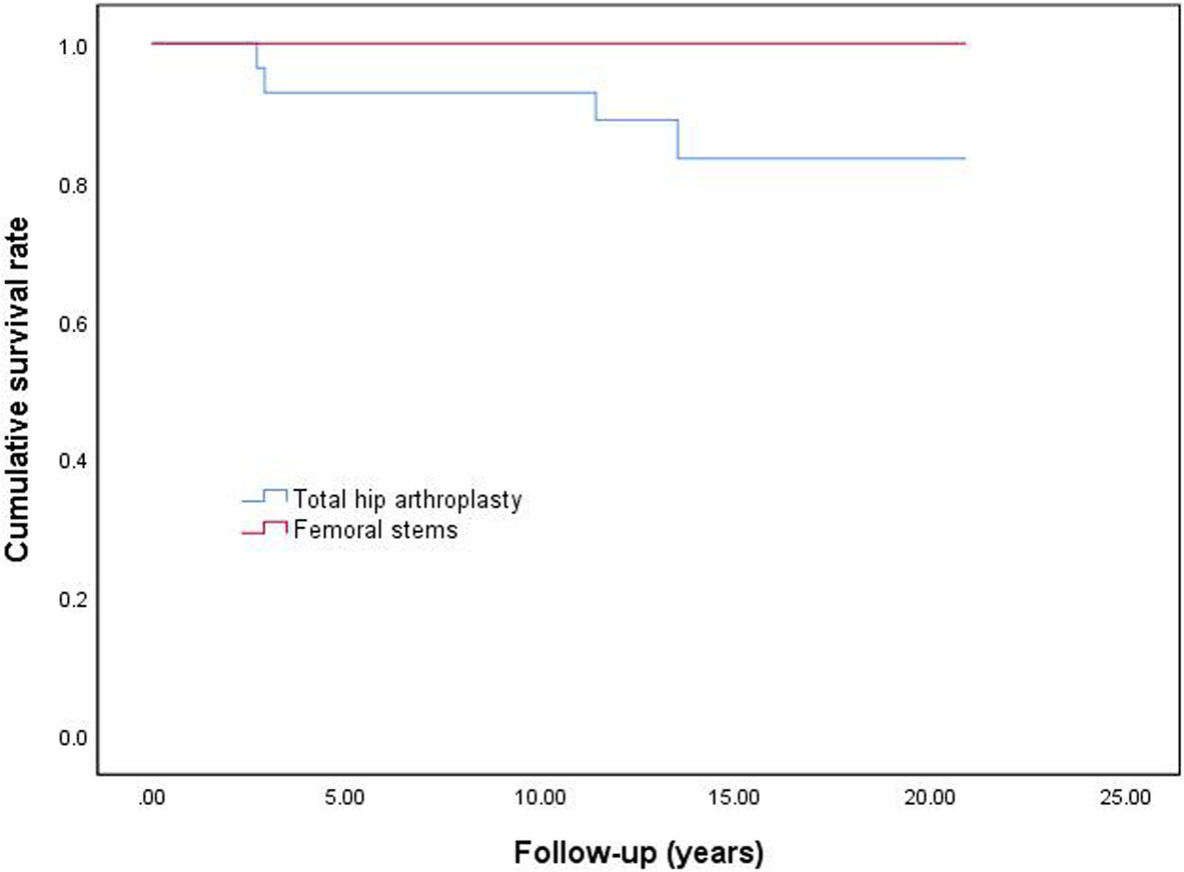
Kaplan–Meier survival curves of survivorship with the endpoint of any revision and only femoral stem revision

Overall, the reoperation rate of THA was 14% (4 out of 28 hips), and 100% patients (28 out of 28 hips) survived the femoral stem procedure. The overall survival rate with the endpoint of any revision was 92.9% (95% confidence interval, 76.5–99.1%) at the 10-year follow-up. Only for the stem as the endpoint of femoral stem revision, a 100% survival rate was noted. The overall expected 15-year survival rate with the endpoint of any revision was 83.4% (95% confidence interval, 67.3–96.0%) and that only for the femoral stem was 100% (Fig. 2).

## Discussion

This study evaluated the midterm outcome of the Wagner cone prosthesis used in femoral revision surgery. Until now, there were no reports on the outcomes of the cementless stem, which was developed for primary THA and used in revision THA. Severe proximal femoral bone loss has been one of the challenging obstacles in successful revision THA. According to the results in this study, the use of the Wagner cone prosthesis could be a reliable treatment strategy in revision THA to achieve satisfactory long-term radiographic and clinical outcomes. This strategy can preserve the bone stock as well as prevent the stress shielding effect in patients undergoing this procedure.

Considering the poor initial outcomes of a cemented stem in revision surgery, a cementless femoral stem is the recommended choice in these cases. The cementless revision stems available in the commercial market are exemplified by the Wagner conical long stem, Link MP, or Zimmer modular stem with various designs including proximal porous-coated, extensively porous-coated, cylindrical, and tapered fluted. All of these stems are longer and fully surface treated with porous coating or grit blasting. These stems can provide an initial firm fixation in the distal portion in various bone defects, especially at the location of a proximal bone defect. However, for the fixation of these stems, more reaming as provided with a long reamer is considered mandatory, meaning that there is a high chance of more pronounced femoral bone loss in this case during revision surgery. Moreover, there is reportedly more proximal stress shielding and more thigh pain associated with this implant [20]. This situation becomes more difficult if the implant is infected or if it fails, as there would be no option for further femoral revision surgery.

The Wagner cone prosthesis is designed for primary THA, especially in patients with femurs characterized with a slim and rather cylindrical configuration and exhibiting a deformation of the intramedullary bony scarring of the proximal end of the femur after previous osteotomies [21, 22]. The Wagner cone prosthesis is fully surface treated with grit blasting that provides and encourages good bone ongrowth in the future. The cone shape design provides distal fixation of the stem, and the 8 ribs provide rotational stability. The stem sizes vary from 13 to 23 with 1 mm increasing thickness that can be used in femurs exhibiting a deformity or a narrow medullary canal. These stem designs can overcome the proximal bone defects in such cases. The large proximal femoral bone defects can be reduced by using a thicker stem after reaming with a round reamer. Even after the process of stem fixation, if there are bone defects that create gaps between the femoral stem and proximal femur, the defect can be filled with morselized bone grafts.

Patients with Paprosky type IIIB or type IV femoral bone loss, who underwent treatment with the extensively porous-coated femoral components, have shown a high rate of mechanical failure [2]. Even though an extensively porous-coated stem for revision THA is reported to have a more than 95% survival rate after a 10-year follow-up, the proximal bone deficiency increased the failure rate in these cases. Furthermore, revision THA in patients with extensive proximal femoral bone loss using a tapered fluted modular femoral component showed a relatively high rate of diaphyseal stress shielding (22%) [23]. In this study, most patients showed satisfactory radiographic and clinical outcomes. Although 3 patients showed subsidence of more than 5 mm, there were no other cases of instability noted during the follow-up after adaptation of the prosthesis to the medullary canal.

With respect to the clinical outcomes, 78.6% of the patients with good or excellent HHS reported minimal or no pain with this process. Thus, considering that thigh pain is a predominant symptom following uncemented femoral revision, it is encouraging that only one patient reported postoperative thigh pain in the latest follow-up. In this study, the total complication rate was 17.8% (Table 3), but the overall expected 15-year survival rate with the endpoint with femoral stem failure was 100%. As compared with previous studies [24–27], we believe that the Wagner cone prosthesis is an acceptable option for revision cases.

**Table 3:** Comparison with other results of femoral revision using femoral stems for primary THA

Years	1994 [25]	1997 [26]	1995 [27]	2001 [28]	
**Authors**	Lawrence	Krishnamurthy	Moreland	Moreland	Present study
**No. of case**	83	297	175	137	28
**F/U duration (years)**	9 (5~13)	8.3 (5~13)	5	9.3 (5~16)	7.6
**Stem design**	AML stem	AML stem	AML stem	AML stem	Wagner cone prosthesis
**Total complication rate**	24 (29%)	5.7% (4%)	19 (10%)	24 (18%)	5 (17.8%)
**Survival**	90%	98.3%	96%	92.7%	100%

To our knowledge, this study is the first to report the long-term outcomes of the Wagner cone prosthesis indicated in revision THA. Compared to revision THA using a longer revision stem, the use of the Wagner cone prosthesis is more advantageous as it has positive patient outcomes in terms of less stress shielding, better preservation of bone stock, less damage to the existing bony structure during revision THA, and low monetary cost. In complicated cases noted frequently, such as those with less bone stock in revision THA, special attention is required during the implant size planning stage. The findings of this study suggest that the Wagner cone prosthesis shows axial and rotational stability when its use is accompanied by adequate bone grafting. However, the implant size should be selected cautiously beforehand, considering the amount of bone defect and existence of compromised bone quality. On the planning template, it is recommended that the contour of the stem should overlap the inner contour of the cortex in the middle third portion of the stem by 1 mm.

**Fig. 3:**
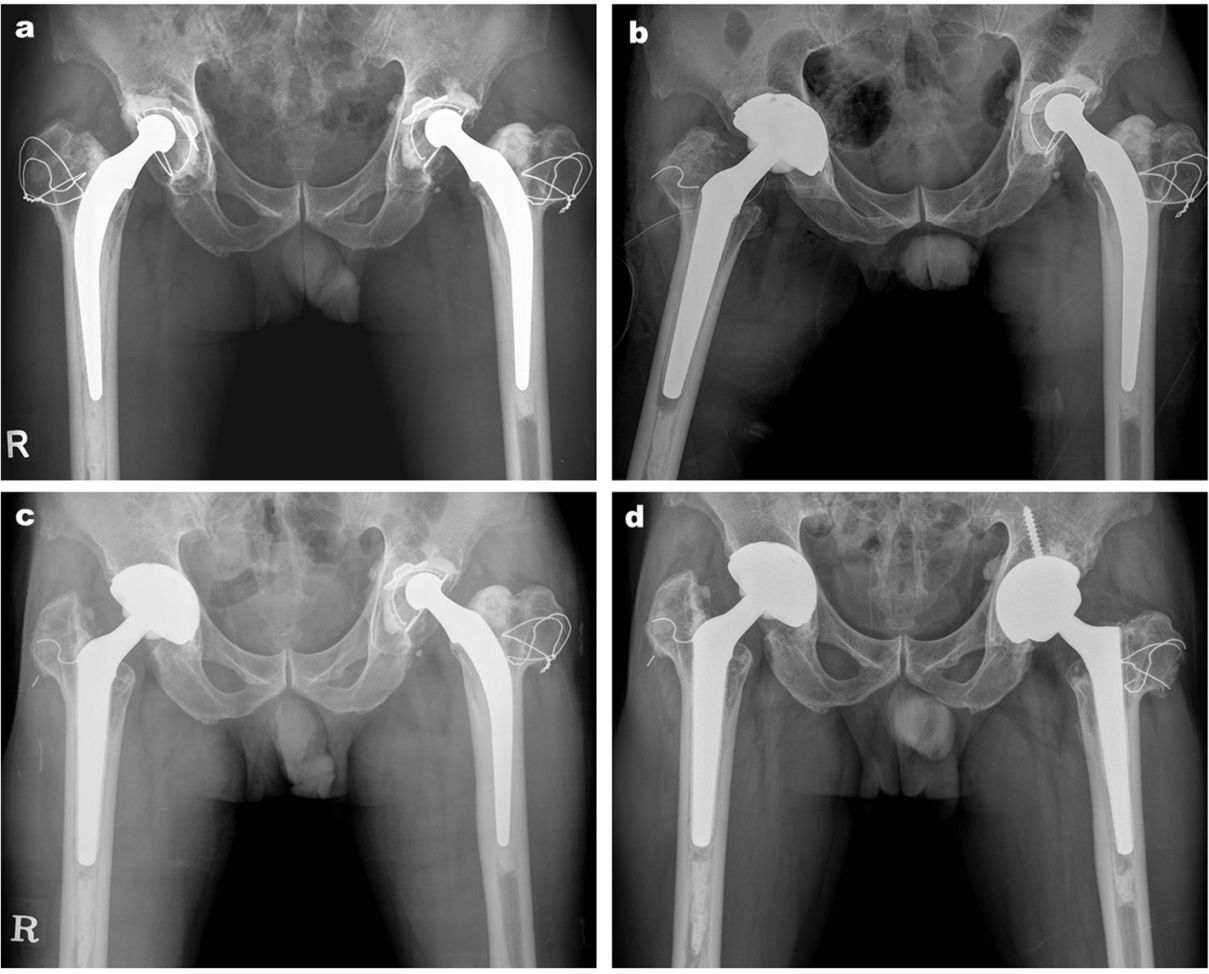
**a** Preoperative radiograph of a 66-year-old male with aseptic loosening of the cemented cup and well-fixed Charnley femoral stem. **b** Immediate postoperative radiograph: revision was performed using the Wagner cone prosthesis and cementless acetabular cup without removing the distal part of the cement. **c** Anteroposterior radiograph of the hip 9 years postoperatively. **d** Anteroposterior radiograph of the hip 15 years postoperatively; the left hip was also revised using the Wagner cone prosthesis 6 years ago

In this study, 5 cemented stems with loosening were subjected to revision surgery using the Wagner cone prosthesis. For the revision from a cemented stem to a cementless stem, cement removal is mandatory, and the most challenging part is the removal of the distal part of the cement. There were 2 hips among 5 hips with remaining cement in the distal part and a cement plug. Because the Wagner cone prosthesis is fixed more proximally to this remnant cement, it was unnecessary to remove the well-fixed distal cement. The other 3 hips showed cement loosening even in the distal part of the cemented stem, which was easy to remove (Fig. 3).

It is known that the incidence of femoral osteolysis after cementless hip arthroplasty increases with time and may progress rapidly. Generally, in femoral stem revision with fully porous-coated implants, continued follow-up and prevention of further osteolysis are essential for optimal patient health outcomes. Therefore, the Wagner cone prosthesis, which guarantees less bone loss during and after surgery, is an appropriate candidate for use in revision THA.

In our series, most patients were relatively young and the bone stock quality was relatively good, thus ensuring favorable outcomes with the Wagner cone prosthesis. However, in the elderly patients and those with poor bone quality, the Wagner self-locking (SL) stem (long length stem) may be a better choice. Zang et al. [28] reported a study of 38 patients (40 hips) who underwent revision THA with the Wagner SL stem. At the last follow-up, incorporation of the grafted bone was observed in 28 hips (70.0%). Sandiford et al. [29] found that the Wagner SL stem could achieve high levels of function with a low revision risk.

Our study has some limitations. First, there was no control group to compare the functional outcomes and clinical outcomes. Second, the sample size was relatively limited. However, as per our knowledge, this report is the first to evaluate the clinical and radiological outcomes of the Wagner cone prosthesis in femoral stem revision.

## Conclusions

In conclusion, we achieved favorable clinical and radiological long-term outcomes in femoral stem revisions using the Wagner cone prosthesis. This cementless femoral stem is a good option for femoral stem revision in cases with relatively good bone stock.

## Data Availability

The data sets supporting the results of this article are included within the article and its additional files. The datasets will be available from the corresponding author on reasonable request.

## Abbreviations

THA: Total hip arthroplasty
HHS: Harris hip score
SL: Self-locking
